# Report of three patients with extensive neurocysticercosis in rural southern Tanzania: neurological, serological and neuroradiological findings

**DOI:** 10.1186/s13256-023-03974-2

**Published:** 2023-07-06

**Authors:** D. Stelzle, C. Makasi, T. M. Welte, C. Ruether, V. Schmidt, S. Gabriel, E. Bottieau, A. Fleury, B. J. Ngowi, A. S. Winkler, the SOLID collaborators, Chiara Trevisan, Inge Van Damme, Pascal Magnussen, Gideon Zulu, Chishala Chabala, Chishimba Mubanga

**Affiliations:** 1 Center for Global Health, Department of Neurology, School of Medicine, Technical University of Munich, Munich, Germany; 2 Muhimbili Medical Research Centre, National Institute for Medical Research, Dar es Salaam, Tanzania; 3 Faculty of Medicine, Kilimanjaro Christian Medical University College, Moshi, Tanzania; 4 Epilepsy Center, Department of Neurology, University Hospital Erlangen, Erlangen, Germany; 5 Department of Neuroradiology, RoMed Clinic Rosenheim, Rosenheim, Germany; 6 Department of Translational Physiology, Infectiology and Public Health, Faculty of Veterinary Medicine, Ghent University, Ghent, Belgium; 7 Department of Clinical Sciences, Institute of Tropical Medicine, Antwerp, Belgium; 8 Departamento de Medicina Genómica y Toxicología ambiental, Instituto de Investigaciones Biomédicas, Universidad Nacional Autónoma de México/Instituto Nacional de Neurología y Neurocirugía, Ciudad de Mexico, Mexico; 9 University of Dar Es Salaam, Mbeya College of Health and Allies Sciences, Mbeya, Tanzania; 10 Department of Community Medicine and Global Health, Institute of Health and Society, Faculty of Medicine, University of Oslo, Oslo, Norway

**Keywords:** Neurocysticercosis, *Taenia solium*, Neglected tropical disease, Epilepsy, Case report

## Abstract

**Background:**

Neurocysticercosis (NCC) is common in eastern Africa, but disease presentation varies considerably. Most patients have single or few NCC-typical lesions in their brain but some present with a large number of lesions. We present three patients with positive antibody-based serology for *Taenia solium* cysticercosis screened at the Vwawa district hospital, Mbozi district, southern Tanzania, in whom extensive NCC was confirmed by neuroimaging.

**Case presentations:**

Patient 1 was a 55-year-old female from the tribe Malila smallholder farmer who has had four generalized tonic–clonic epileptic seizures over a period of 11 years and one episode of transient left hemiparesis one year before seizure onset. The patient also reported monthly to weekly episodes of severe, progressive, unilateral headache. The computed tomography (CT) scan of the brain showed 25 NCC lesions of which 15 were in the vesicular stage. Patient 2 was a 30-year-old male from tribe Nyha mechanic who reported monthly episodes of moderate to severe, progressive, bilateral headache, but no epileptic seizures. The CT scan showed 63 NCC lesions of which 50 were in the vesicular stage. Patient 3 was a 54-year-old female from the tribe Malila smallholder farmer who suffered from frequent generalized tonic–clonic epileptic seizures with potential signs of focal seizure onset. She also reported weekly to daily episodes of severe, progressive, unilateral headache. The CT scan showed 29 NCC lesions of which 28 were in the vesicular stage.

**Conclusions:**

Clinical presentation of NCC with multiple brain lesions varies considerably ranging from few epileptic seizures and severe headache to severe epilepsy with frequent epileptic seizures. Individuals with neurological signs/symptoms that may be due to NCC, based for example on epidemiological criteria or serological evidence of cysticercosis, are recommended to undergo neuroimaging before anthelminthic treatment is considered.

## Background

Neurocysticercosis (NCC) is the most common helminthic disease of the central nervous system, accounting for up to every third case of epilepsy in regions endemic for the pork tapeworm *Taenia solium* [1–5]. Prevalence is highest in low-income and middle-income countries but varies between world regions. It is more common in rural areas, especially where pigs roam freely and sanitary conditions are poor.

Despite many endemic areas for *T. solium*, in Africa knowledge about the disease is limited, even among health workers [6, 7]. Even though many people know about cyst-infected pork, the link to human disease is seldomly known. This could be one of the reasons—together with the lack of affordable diagnostic tests and limited access to neuroimaging—why NCC is seldomly or never diagnosed in Africa.

NCC often remains silent for years but when symptomatic, NCC commonly presents with epileptic seizures or severe headache, but sometimes also with focal neurologic deficits, altered mental status, or psychotic episodes. Signs and symptoms depend on the number, location and the stage of the lesion [8–10]. Symptoms most commonly develop in patients with degenerating cysts (colloidal-vesicular or granular-nodular stage). Whilst patients with parenchymal cysts more commonly present with seizures (especially when cysts are located near the cortex), patients with extraparenchymal cysts more often present with headache episodes [11, 12]. Focal neurological signs/symptoms including hemiparesis can occur, which may be due to the location of the cerebral or spinal NCC cyst or result from NCC-induced vasculitis [13, 14]. In this case series, we highlight that NCC, even when associated with high parasitic load, can present in different ways, posing a challenge for treatment decisions for which no guidelines are in place.

## Case presentations

We present three patients with extensive NCC lesions and varied clinical presentations. Patients were recruited as part of the project SOLID (Pan African Clinical Trials Registry: PACTR201712002788898) which aimed to evaluate a novel point-of-care test (POC test) for the detection of *T. solium* cysticercosis and taeniosis antibodies [15, 16]. Within that project, patients with and without neurological signs/symptoms of NCC (epileptic seizures and/or severe headache) were recruited through mental health clinics and out-patient departments of three district hospitals in southern Tanzania. All patients were tested with the novel POC test and, if positive for cysticercosis or taeniosis, the patient underwent further clinical work-up, including reference testing and neuroimaging. Furthermore, every tenth mental health clinic patient with negative POC for cysticercosis and taeniosis underwent further examination. The POC test is a lateral flow assay and contains two strips; one with recombinant rES33 antigen for the diagnosis of taeniosis and one with the recombinant rT24h antigen for the detection of cysticercosis antibodies. Confirmatory reference tests for antibodies comprise the lentil lectin-bound glycoproteins-enzyme-linked immunoelectrotransfer blot (LLGP-EITB), which is the test of choice for cysticercosis antibody detection, as well as the rES33 and rT24h immunoblots. For the detection of cysticerci antigens in serum, the B158/B60 antigen ELISA is commonly performed. Other methods, such as the copro-antigen ELISA and polymerase chain reaction (PCR) techniques in stool for the detection of *T. solium* antigens are available, but mostly used in research settings as laboratories in resource-constrained areas lack equipment and infrastructure [17]. In the SOLID project all of the above-mentioned tests were used. All serological tests have imperfect diagnostic accuracies, for example because of cross-reactions with other helminths.

### Patient 1

**Fig. 1:**
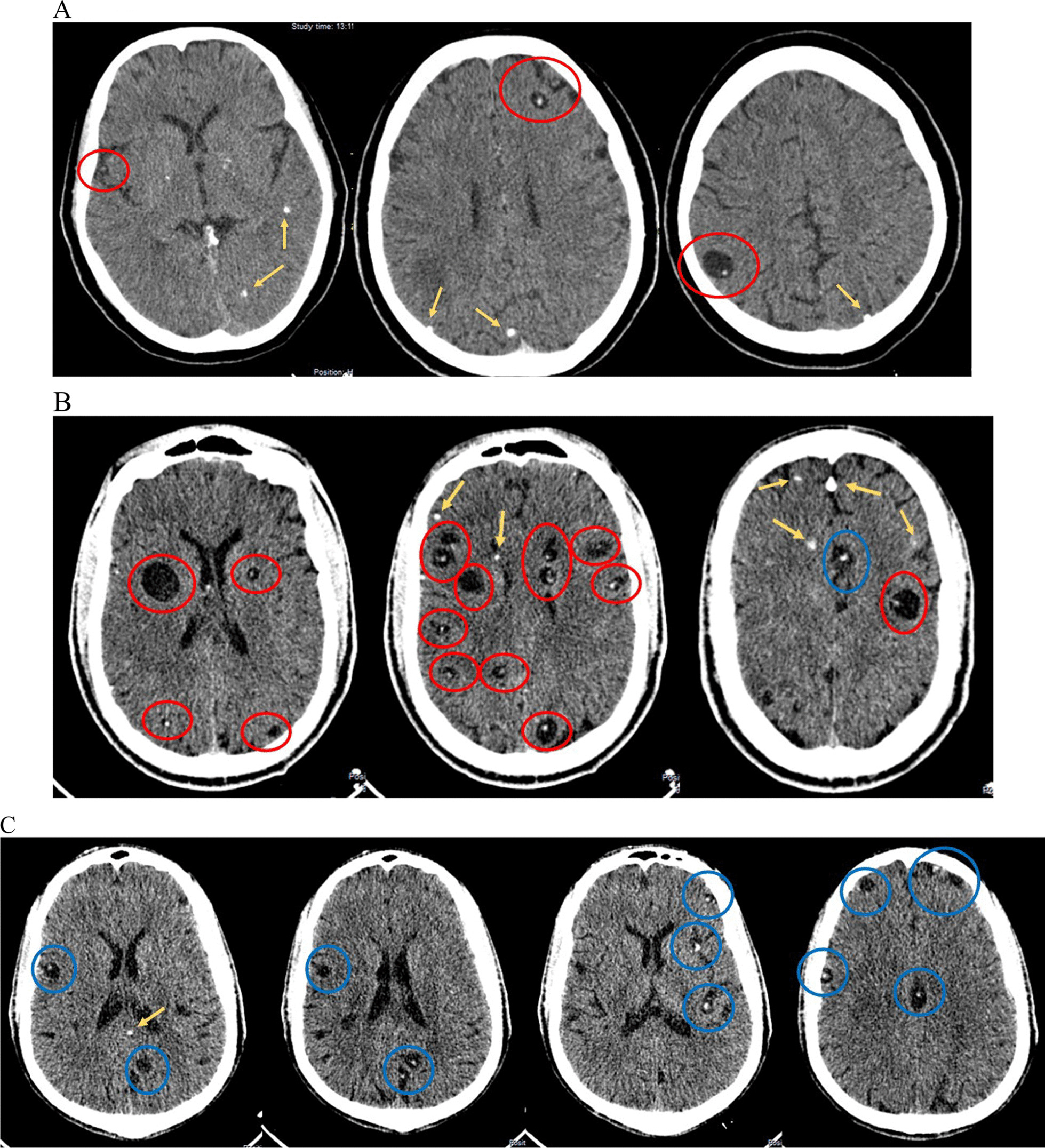
**A** Patient 1: Calcifications in the occipital lobe bilaterally, left temporal lobe and bilaterally in the basal ganglia; in addition there are various cystic NCC lesions, two of them showing a hyperdense core, which represents the scolex (= head of the larvae); **B** Patient 2: A large number of vesicular and calcified lesions scattered throughout the brain; cysts are of varying size, some of them showing a scolex; **C** Patient 3: Extraparenchymal vesicular lesions which are found in the subarachnoid space in the sulci and in the interhemispheric fissure. Red circle: Viable lesions (with and without scolex) in the parenchyma. Blue circle: Viable lesions (with and without scolex) in the extraparenchymal space. Yellow arrow: Calcified lesions

This 55-year-old female from the tribe Malila was included into the SOLID project because she presented with nausea and loss of appetite at the outpatient department (Table 1). Additionally, the patient screened positive for epileptic seizures with report of four generalized tonic–clonic epileptic seizures over the course of 11 years. Onset was reported at the age of 44 and the last seizure occurred six months prior to CT scan. Furthermore, she complained about monthly to weekly episodes of severe, progressive, throbbing/pulsating headache located on the right side of the forehead and the frontal part of the head. Headache episodes lasted for several days, were accompanied by photophobia and blurred vision and were relieved by paracetamol. The patient reported hemiparesis one year before the first epileptic seizure which had resolved within three days. No other non-communicable diseases were reported. Neurological examination did not show any pathological findings. The POC test was positive for cysticercosis and so was the rT24h immunoblot and the serum antigen ELISA. LLGP-EITB and the tests for *T. solium* taeniosis were negative. The CT scan showed overall 25 lesions, 23 in the brain parenchyma (14 in active stage of which one showed perilesional oedema, 9 calcifications) and 2 in the extraparenchymal space of which one was located in the right Sylvian fissure (Fig. 1A). The patient was initiated on 2 × 400 mg carbamazepine per day as she had not been on anti-seizure medication (ASM) before and experienced more than one seizure in the previous two years. No anthelminthic medication was administered because of the large number of lesions. At follow-up after 6 months, the patient who was under continued ASM did not report any further epileptic seizures. In the CT scan, the one cyst in the parietal lobe resolved including the perilesional oedema.

**Table 1:** Characteristics of patients with active neurocysticercosis

	Patient 1	Patient 2	Patient 3
**Gender**	Female	Male	Female
**Age**	55 years	30 years	54 years
**Occupation**	Smallholder farmer	Mechanic	Smallholder farmer
**Epileptic seizures**	Yes	No	Yes
Type of epileptic seizure	Generalized tonic–clonic	–	Generalized tonic–clonic and potential focal unaware seizures with rolling eyes and grinding of teeth
Impaired consciousness/ loss of consciousness	Yes, from the beginning	–	Yes, from the beginning
Frequency	Only four epileptic seizures in 11 years The last two within 2 years	–	No seizures since on phenytoin (6 months) before ~ 3 per month
Accompanying symptoms	Tongue bite, froth from the mouth	–	Tongue bite, froth from the mouth
Reorientation phase	No	–	Yes, 30 min terminal sleep
Age at first seizure	44	–	25
Anti-seizure medication	Patient was initiated on carbamazepine 2 × 400 mg/d	–	Phenytoin 100 mg/d
Family history of epilepsy	No	No	No
**Headache**	Yes	Yes	Yes
Type of headache	Progressive headache	Progressive headache	Progressive headache
Side of headache	Unilateral (right side)	Bilateral	Unilateral (left side)
Location	Forehead, frontal part of head	Forehead	Forehead, frontal part of head
Intensity	4/5	3/5	4/5
Quality	Throbbing, pulsating	Throbbing, pulsating	Throbbing, pulsating
Duration	Several days	Several hours	Less than 1 h
Frequency	Monthly to weekly	Monthly	Weekly to daily
Accompanying symptoms	Photophobia, blurred vision	Photophobia, nausea, vomiting	-
**Neurological examination‡**	No pathological finding	No pathological finding	No pathological finding
**Other medical condition**	Not currently, but patient reported sudden onset left hemiparesis which resolved within 3 days (one year before seizures started)	No	No
**Point-of-care (POC) test**	Cysticercosis positive, taeniosis negative	Cysticercosis positive, taeniosis negative	Cysticercosis positive, taeniosis negative
**Serological testing**			
LLGP-EITB	Negative	Negative	Positive
rES33 immunoblot	Negative	Negative	Positive
rT24h immunoblot	Positive	Positive	Positive
Antigen ELISA	Positive	Positive	Positive
Stool testing			
Copro-antigen ELISA	Negative	Negative	Negative
**CT findings**			
Number of lesions	25	63	29
Vesicular lesions	*15/25*	*50/63*	*28/29*
Colloidal-vesicular lesions	*1/25*	*–*	*–*
Granular-nodular lesions	*–*	*–*	*–*
Calcified lesions	*9/25*	*13/63*	*1/29*
Parenchymal lesions	23	49	15
Active stage lesions^†^	*14/23*	*36/49*	*14/15*
Calcified lesions	*9/23*	*13/49*	*1/15*
Extraparenchymal lesions^£^	2	14	14
Active stage lesions	*2/2*	*14/14*	*14/14*
Calcified lesions	*–*	*–*	*–*
Hydrocephalus	No	No	No
**Follow-up after 6 months**			
Symptoms	Patient has not had any seizures since last visit	No change, patient has not developed seizures, but headache persists	Patient has not had any seizures since last visit
CT findings	1 active parenchymal lesion with perilesional oedema resolved	No change	1 active parenchymal lesion resolved

^†^Vesicular, colloidal-vesicular and granular-nodular stage are considered active stage lesions; ^£^all lesions were in the subarachnoid space around the cortex or in the Sylvian fissure; none were intraventricular or in the basal cisterns; ^‡^examined were cranial nerves, muscle strength, muscle tone, tendon reflexes, extrapyramidal system, coordination and gait, sensory system, and mental state

### Patient 2

This 30-year-old male from tribe Nyha was recruited from the outpatient department because of headache and abdominal discomfort. The patient did not report any seizures, but monthly episodes of moderate to severe, progressive, throbbing/pulsating headache, located bilaterally on the forehead (Table 1). Headache episodes lasted for several days and were accompanied with photophobia, nausea and vomiting. The patient mentioned vomiting as a mechanism for headache relief, but otherwise episodes terminated by themselves. There was no past medical history and neurological examination did not show any pathological results. The POC test was positive for cysticercosis, and so were the rT24h immunoblot and the serum antigen ELISA. The LLGP-EITB was negative. The diagnostic tests for *T. solium* taeniosis were also negative. The CT scan showed overall 63 lesions, 49 in the brain parenchyma (36 in active stage and 13 calcifications) and 14 in the extraparenchymal space (Fig. 1B). No preventive ASM therapy was administered, and no specific anthelminthic therapy because of the location and number of lesions. At follow-up after 6 months, no change was seen in the CT scan and the patient had not developed any seizures.

### Patient 3

This 54-year-old female from the tribe Malila was included in SOLID because she regularly attended mental health clinics for epilepsy and used to suffer from approximately 3 seizures per month with reported signs of potential focal seizure onset (unawareness and grinding of teeth) but also generalized tonic–clonic seizures (Table 1). Furthermore, she also complained about severe, progressive, throbbing/pulsating headache located on the left side of the forehead and the frontal part of the head. Headache episodes lasted for less than one hour but occurred very often (weekly to daily). No non-communicable disease was reported, and the neurological examination did not show any pathological findings. The POC test was positive for cysticercosis and so were the serological reference tests (LLGP-EITB, rT24h immunoblot and serum antigen ELISA). The diagnostic tests for *T. solium* taeniosis were negative. The CT scan showed 29 lesions overall, 15 in the brain parenchyma (14 in active stage, 1 calcification) and 14 in the extraparenchymal space (Fig. 1C). At the time of recruitment, the patient had been on phenytoin 100 mg per day for 6 months, and seizure free since then, despite the low dosage of ASM. No specific anthelminthic therapy was initiated because of the large number of lesions. At follow-up after 6 months, the patient did not report any further epileptic seizures. In the CT scan, the one cyst in the parietal lobe resolved including the oedema.

## Discussion

Depending on the infection pressure and other factors, NCC can present radiologically in various ways from single calcified lesions to multiple active stage lesions [18]. As we showed, even among patients with extensive NCC, clinical presentation varies considerably from mildly affected with no or only few epileptic seizures to highly symptomatic with frequent epileptic seizures and severe headache episodes. Interestingly, many patients even with large number of lesions do not show any pathological neurological signs—at least no obvious or persistent ones [8]. In our cohort, Patient 1 reported a transient hemiparesis which has been described as a sign of NCC and may have been due to transient perilesional oedema. Mental state alterations or cognitive decline may also arise, especially in extensive NCC, but are often overlooked, if not severe [19, 20]. Within the SOLID project, a community-based study was conducted in Zambia which found that less than every fifth patient with NCC had any neurological signs/symptoms (data unpublished).

Seizures were reported by two patients (number 1 and 3) who had several lesions in the brain parenchyma (23/25 and 15/29), whereas patient 2, who had the most parenchymal lesions (49/63), did not experience any epileptic seizures. This underlines the fact that seizure generation in the presence of NCC seems to be multifactorial and not only dependent on the number of parenchymal lesions. Signs of focal seizure onset, as is typical for NCC, were only present in patient 3, with a suspected temporal seizure semiology (unaware seizures with grinding of teeth), either from a temporal lesion or as a result of propagation from a neighbouring symptomatic lesion. Nevertheless, assessment of focal seizures often is difficult in sub-Saharan Africa due to language barriers and unawareness of neurological sigs/symptoms pertaining to epileptic seizures [21]. This also complicates assessment of treatment success. Yet, both patients with epileptic seizures seemed to respond well to ASM, which is in line with findings of a previous study [22]. Nevertheless, these two patients also demonstrate the need for optimization of ASM therapy in people with epilepsy who do not have a continuous supply of medication and no specialized neurological workforce for example adequate dosage advice [23].

Patient 2 did not experience epileptic seizures, but the main symptoms were headache, nausea and vomiting. This is more consistent with clinical manifestation of extraparenchymal NCC [12]. Indeed, this patient showed 14 extraparenchymal cysts, mainly located in the Sylvian fissure. Interestingly, the 49 parenchymal lesions did not appear to have caused any (focal) neurological signs/symptoms including epileptic seizures at the time of presentation but certainly carry a risk for the future occurrence of those signs/symptoms. The question on preventive ASM in those cases is not easy to answer. So far, no studies have been conducted on the preventive use of ASM for primary seizure control in NCC patients [24]. Data are scarce but general consensus is that only around 20% of people with NCC will develop epileptic seizures in their life time—usually at the time when cysts degenerate and an inflammatory reaction is produced by the host. Hence, the more lesions someone has, the more chances there are for epileptic seizures to occur at degeneration of these lesions. Moreover, calcifications may also act as foci for epileptic seizures and therefore cause (chronic) epilepsy. However, in one community-based study in Ecuador only 9 of 118 patients (7.6%) with calcified NCC had epilepsy [25]. It is also hypothesized, that epileptic seizures based on calcifications may only develop temporarily, especially in the context of perilesional oedema [26]. The reason why perilesional oedema may develop around some calcified lesions is still not clear, but the most likely explanation is that the oedema represents an inflammatory response to antigen sporadically released from calcified granulomas [27]. We initially considered ASM as primary prevention for our patient but ultimately opted for an enhanced surveillance at district hospital. The reasons were the cost of ASM, the common stock-outs, which may cause withdrawal seizures, but also stigma and discrimination that are still associated with epilepsy and its treatment in many parts of Tanzania [6, 23, 28, 29]. The most important in those patients is to inform them and their relatives regarding the risk that epileptic seizures may occur in the context of NCC, the type of headache that may be associated with NCC and the necessity to seek medical advice if any relevant neurological signs/symptoms should appear. Also, it needs to be stated that the occurrence of headache associated with NCC most likely is multifactorial and may be due to degenerating parenchymal cysts triggering an inflammatory response, extraparenchymal NCC causing chronic inflammation and, not the least, coincidental primary headache syndromes such as migraine and tension-type headache. In rare cases, the headache may also indicate an increase in intracranial pressure due to intraventricular NCC.

Considerations for anthelminthic treatment are as controversial as ASM for primary prevention of epileptic seizures in patients with extensive NCC. In general, anthelminthic treatment of NCC in sub-Saharan Africa is complicated because of the lack of medication (anthelminthics and appropriate corticosteroids), neuroimaging facilities, intensive care units and the possibility to properly monitor a patient with extensive NCC in case of therapy-related adverse events such as status epilepticus or increased intracranial pressure. Furthermore, treatment in Africa may be challenged by HIV co-infection which may predispose to more side effects and alterations of the efficacy of anthelminthic medication. Furthermore, corticosteroid therapy may have to be adjusted because blood glucose levels and blood pressure are less stable among people living with HIV [30, 31]. WHO guidelines on management of NCC in low-resource settings recommend treatment with either albendazole or praziquantel, always in combination with corticosteroids, depending on the availability [32]. So far, only one study in Africa has assessed differences in treatment efficacy and adverse events of monotherapy with albendazole and combination therapy with albendazole and praziquantel [33]. The latter is recommended by the Infectious Diseases Society of America (IDSA) and the American Society of Tropical Medicine and Hygiene (ASTMH) for patients with at least 3 vesicular NCC lesions [33].

For treatment decision in the 3 presented cases, it is important to consider the severity of neurological signs/symptoms. Our 3 patients had between 15 and 50 vesicular lesions but did not show any pathology on neurological examination and seizures were controllable by ASM, if administered regularly. Hence, we concluded it would not be reasonable to treat these patients with anthelminthic medication carrying the risk of exacerbation of neurological signs/symptoms due to degeneration of cysts. Continued symptomatic therapy and a clinical follow-up every half year to one year seemed more appropriate—ideally with neuroimaging, but outside research access to neuroimaging seems unlikely in Tanzania. Additionally, our patients and their relatives were provided with information about NCC and potentially associated neurological signs/symptoms and advised to seek medical care, should any of those signs/symptoms occur. Considering patient 2 with the leading clinical presentation of extraparenchymal NCC, an examination of the cerebrospinal fluid (CSF) should be considered in the further clinical work up, as cysts in the Sylvian fissure, similar to cysts in the basal cisterns or in the ventricles, may be associated with inflammatory CSF, and the ensuing risk of hydrocephalus, vasculitis and/or arachnoiditis [12]. If inflammation is present, initiation of anthelminthic treatment could be considered, as a persistent inflammatory CNS syndrome carries a significant risk for further complications [12, 34].

## Conclusion

In summary, we present an interesting case series of 3 patients with extensive NCC and different neurological signs/symptoms that opens up for new questions on the best management of this patient group in low-resource settings and additionally highlights the persisting lack of knowledge, data, diagnostic and therapeutic possibilities in the context of NCC in sub-Saharan Africa. Unfortunately, data on further clinical investigations such as electroencephalography, cerebrospinal fluid analysis and cerebral magnetic resonance imaging were not available, which could have provided further insight, especially into the presence and distribution of extraparenchymal NCC. Our case series also demonstrated the variability of neurological and other clinical signs/symptoms in the context of NCC. Headache associated with NCC is most likely multifactorial and largely unexplored but could provide important clues to pathology of NCC and the resulting management options. The heterogeneity of clinical and in particular neurological signs/symptoms in the context of NCC makes neuroimaging a “conditio since qua non” when anthelminthic treatment is considered and should be further prompted by the presence of epidemiological criteria and serological evidence of cysticercosis, if available. As neuroimaging is not always accessible in resource-constrained setting, symptomatic management, for example with ASM, close clinical follow-up and good communication with patients and their relatives about the risks of NCC represent an alternative for management of NCC.

## Data Availability

All data collected are presented in the case reports.

## Abbreviations

ASTMH: American Society of Tropical Medicine and Hygiene
ASM: Anti-seizure medication
CNS: Central nervous system
CSF: Cerebrospinal fluid
CT: Computed tomography
ELISA: Enzyme-linked immunosorbent assay
HIV: Human immunodeficiency virus
IDSA: Infectious Diseases Society of America
LLGP-EITB: Lentil lectin-bound glycoproteins/enzyme-linked immunoelectrotransfer blot
NCC: Neurocysticercosis
PCR: Polymerase chain reaction
POC test: Point-of-care test
rES33: Protein name
rT24h: Protein name
SOLID: Project on the evaluation of a diagnostic point-of-care test for (neuro)cysticercosis
*T. solium*: *Taenia solium*
WHO: World Health Organization
